# Pulmonary congestion during Exercise stress Echocardiography in Hypertrophic Cardiomyopathy

**DOI:** 10.1007/s10554-022-02620-0

**Published:** 2022-11-02

**Authors:** Eszter Dalma Pálinkás, Federica Re, Jesus Peteiro, Milorad Tesic, Attila Pálinkás, Marco Antonio Rodrigues Torres, Ana Djordjevic Dikic, Branko Beleslin, Caroline M. Van De Heyning, Maria Grazia D’Alfonso, Fabio Mori, Quirino Ciampi, José Luis de Castro Silva Pretto, Iana Simova, Viktória Nagy, Krisztina Boda, Róbert Sepp, Iacopo Olivotto, Patricia A. Pellikka, Eugenio Picano

**Affiliations:** 1 Doctoral School of Clinical Medicine, University of Szeged, Szeged, Hungary; 2 Division of Non-Invasive Cardiology, Department of Internal Medicine, Albert Szent- Györgyi Clinical Center, University of Szeged, Szeged, Hungary; 3 Cardiology Department, San Camillo-Forlanini Hospital, Rome, Italy; 4 CHUAC and University of A Coruña, CIBER-CV A Coruña, Spain; 5 Cardiology Clinic, Medical School, University Center Serbia, University of Belgrade, Belgrade, Serbia; 6 Internal Medicine Department, Elisabeth Hospital, Hódmezővásárhely, Hungary; 7 Federal University of Rio Grande do Sul, Porto Alegre, Brazil; 8 Department of Cardiology, Antwerp University Hospital, Edegem, Belgium; 9 Cardiovascular Imaging Division, Careggi University Hospital, Florence, Italy; 10 Fatebenefratelli Hospital, Benevento, Italy; 11 Hospital Sao Vicente de Paulo e Hospital de Cidade, Passo Fundo, Brazil; 12 Heart and Brain Center of Excellence, University Hospital, Pleven, Bulgaria; 13 Department of Medical Physics and Informatics, University of Szeged, Szeged, Hungary; 14 Cardiomyopathy Unit, Careggi University Hospital, Florence, Italy; 15 Department of Cardiovascular Medicine, Mayo Clinic, Rochester, Minnesota USA; 16 Institute of Clinical Physiology, CNR, Pisa, Italy

**Keywords:** B-lines, Pulmonary congestion, Hypertrophic cardiomyopathy, Exercise stress echocardiography, Echocardiography

## Abstract

**Background:**

B-lines detected by lung ultrasound (LUS) during exercise stress echocardiography (ESE), indicating pulmonary congestion, have not been systematically evaluated in patients with hypertrophic cardiomyopathy (HCM).

**Aim:**

To assess the clinical, anatomical and functional correlates of pulmonary congestion elicited by exercise in HCM.

**Methods:**

We enrolled 128 HCM patients (age 52 ± 15 years, 72 males) consecutively referred for ESE (treadmill in 46, bicycle in 82 patients) in 10 quality-controlled centers from 7 countries (Belgium, Brazil, Bulgaria, Hungary, Italy, Serbia, Spain). ESE assessment at rest and peak stress included: mitral regurgitation (MR, score from 0 to 3); E/e’; systolic pulmonary arterial pressure (SPAP) and end-diastolic volume (EDV). Change from rest to stress was calculated for each variable. Reduced preload reserve was defined by a decrease in EDV during exercise. B-lines at rest and at peak exercise were assessed by lung ultrasound with the 4-site simplified scan. B-lines positivity was considered if the sum of detected B-lines was ≥ 2.

**Results:**

LUS was feasible in all subjects. B-lines were present in 13 patients at rest and in 38 during stress (10 vs 30%, p < 0.0001). When compared to patients without stress B-lines (n = 90), patients with B-lines (n = 38) had higher resting E/e’ (14 ± 6 vs. 11 ± 4, p = 0.016) and SPAP (33 ± 10 vs. 27 ± 7 mm Hg p = 0.002). At peak exercise, patients with B-lines had higher peak E/e’ (17 ± 6 vs. 13 ± 5 p = 0.003) and stress SPAP (55 ± 18 vs. 40 ± 12 mm Hg p < 0.0001), reduced preload reserve (68 vs. 30%, p = 0.001) and an increase in MR (42 vs. 17%, p = 0.013) compared to patients without congestion. Among baseline parameters, the number of B-lines and SPAP were the only independent predictors of exercise pulmonary congestion.

**Conclusions:**

Two-thirds of HCM patients who develop pulmonary congestion on exercise had no evidence of B-lines at rest. Diastolic impairment and mitral regurgitation were key determinants of pulmonary congestion during ESE. These findings underscore the importance of evaluating hemodynamic stability by physiological stress in HCM, particularly in the presence of unexplained symptoms and functional limitation.

## Introduction

Hypertrophic cardiomyopathy (HCM) is the most common genetic disorder of the myocardium with variable phenotypic expression (1). Exploration of new clinical markers related to cardiac pathophysiology through the prism of cardiac imaging may help to identify the functional heterogeneity and different phenotypes (2), which represent potential therapeutic targets in HCM (3). Current European guidelines assign IB class of recommendations to exercise stress echocardiography (ESE) in symptomatic HCM patients without resting left ventricular outflow tract (LVOT) obstruction, to detect hemodynamically important exercise-induced LVOT gradient (LVOTG) and mitral regurgitation (MR) (4). However, the information provided by ESE in HCM extends far beyond the evaluation of the LVOTG and MR (5, 6). B-lines can be assessed by lung ultrasound (LUS) during ESE and provide a unique way to evaluate semi-quantitatively extra-vascular lung water, a physiologic variable with well-established diagnostic and prognostic value in a range of cardiac diseases (7). B-lines at rest and during stress in HCM may help to identify the pulmonary congestion phenotype, which is an actionable therapeutic target for diuretic therapy. Proper use of diuretics is a challenging issue in HCM, as these agents may decrease preload and worsen dynamic obstruction if used inappropriately. Despite the growing evidence on the clinical significance of exercise-induced pulmonary congestion assessment by LUS, its clinical value has never been investigated in patients with HCM. Therefore, in this study we aimed to evaluate the feasibility of stress LUS in HCM and to assess the clinical, anatomical and functional correlates of pulmonary congestion during ESE in HCM.

## Methods

### Study population

We enrolled 128 consecutive HCM patients from 10 different SE laboratories [Rome, Italy (n = 54); Belgrade, Serbia (n = 17); Szeged - Hodmezovasarhely, Hungary (n = 17); A Coruna, Spain (n = 14); Porto Alegre, Brazil (n = 12); Antwerp, Belgium (n = 6); Florence, Italy (n = 4); Benevento, Italy (n = 2); Passo Fundo, Brazil (n = 1); Pleven, Bulgaria (n = 1)] of the Stress echo 2020 multicenter study (8). Diagnosis of HCM was based on the contemporary guidelines cautiously excluding HCM phenocopies (4). All patients underwent symptom-limited dynamic echocardiographic examination according to the referring physician’s indications as part of the routine work-up. The inclusion criteria were: (1) Diagnosis of HCM; (2) age > 18 years; (3) no known coronary artery disease; (4) ability to perform ESE. The following exclusion criteria were used: (1) comorbidities known to generate B-lines of extracardiac origin (e.g. pulmonary fibrosis, lung cancer, pneumonia); (2) atrial fibrillation; (3) technically poor acoustic window precluding sufficient imaging of the left ventricle (LV); (4) resting ejection fraction (EF) < 40%, (5) HCM phenocopies of non-sarcomeric nature (Fabry, Danon and amyloidosis). The study was conducted in accordance with the Declaration of Helsinki. The study protocol and the informed consent were reviewed and approved by the institutional ethics committees as a part of the SE 2020 study. All subjects gave their informed consent for inclusion before they participated in the study. Sudden cardiac death risk (SCD) was determined according to the European Society of Cardiology’s HCM Risk-SCD formula (4).

### Exercise stress

Patients underwent ESE according to the recommended protocols with one of the following stresses: semi-supine bicycle (25 watts increments every 2 or 3 min); upright bicycle; treadmill exercise with modified Bruce protocol (9). Routinely used medications were administered as usual before and after the exam. Electrocardiogram and blood pressure were monitored continuously. Criteria for terminating the test were severe chest pain, diagnostic ST-segment shift, excessive blood pressure increase [systolic blood pressure (SBP) ≥ 240 mmHg, diastolic blood pressure ≥ 120 mmHg], symptomatic hypotension with a sudden drop in blood pressure (> 40 mmHg), limiting dyspnea, maximal predicted heart rate (HR), significant arrhythmias or limiting side effects (7, 8).

### Hemodynamic measurements

All echocardiographic measurements were measured at rest and with stress by experienced cardiologists according to standard criteria of execution and interpretation recommended by the American Society of Echocardiography and the European Association of Cardiovascular Imaging (9, 10, 11). Wall motion score index (WMSI) was calculated applying the four-point score system ranging from 1 (normal) to 4 (dyskinetic) in a 17-segment model of the left ventricle. LV volumes were evaluated by the biplane Simpson method. LVOTG was the maximum instantaneous gradient as measured by continuous-wave Doppler. LV force was defined by the following formula: LVOTG + SBP / end-systolic volume (ESV). LV contractile reserve was calculated by dividing the stress by rest LV force values. Heart rate reserve was calculated as the peak/rest HR from 12-lead ECG (7). Stroke volume (SV) was calculated as end-diastolic volume (EDV)-ESV. Cardiac output was computed using the following formula: EDV-ESV x HR. Cardiac output and SV were normalized to body surface area to obtain SV index and cardiac index (CI). Preload reserve impairment was defined as peak stress EDV < rest EDV (12). MR was evaluated with semi-quantitative method and graded as: none or trivial (0), mild (1), moderate (2), and severe (3) (13). Pulse pressure was assessed by the difference between SBP and diastolic blood pressure. Abnormal blood pressure response was defined as the fall of SBP by > 20 mm Hg or a failure to increase the SBP by > 20 mm Hg during exercise (4). The ESE examinations were performed by cardiologists who were not involved in the patients’ management and had passed the quality control procedures upstream to patient recruitment, with inter-observer variability < 10% in quantifying B-lines and < 10% in estimating LV area by planimetric method (7, 8, 14).

### Lung ultrasound

The LUS acquisition was performed at rest and peak (or immediately after) stress with the 4-site simplified scan at the third intercostal space on the anterior and lateral hemithoraces, using the same probe employed for the cardiac scan. B-lines were defined as hyperechoic reverberation artifacts rising from the pleural line to the bottom of the screen moving synchronously with lung sliding without fading (7). After scanning the 4 chest sites, the cumulative B-line score was obtained by summing the number of detected B-lines at each site. B-lines were considered present if at least 2 B-lines could be detected.

### Statistical analysis

Continuous variables were expressed as mean ± standard deviation or median and IQR, according to the variable’s distribution. Categorical variables were reported as frequency and percentage. Data distribution was assessed graphically. Student’s independent t-test and Mann–Whitney U test were used to compare differences between continuous variables. Categorical variables were compared using Chi-squared test or Fisher’s exact test. Spearman’s correlation was used to assess the relationship between stress B-lines and functional parameters. Univariate and multivariate logistic regression analyses were performed to assess the baseline predictors of exercise B-lines. The multivariate analysis was performed on clinically relevant variables with forward stepwise method using likelihood ratio test. Statistical significance was set at p < 0.05. Statistical Package for the Social Sciences (IBM SPSS Statistics, version 26) and MedCalc for Windows (version 7.6.0.0.) was employed for analysis.

## Results

### Baseline characteristics

In a total of 128 HCM patients (age 50 ± 15 years, 85 men) LUS and echocardiographic examinations were performed applying bicycle in 82 (64%; semi-supine in 28 and upright in 54) and treadmill in 46 (36%) with LV imaging at peak stress or in the immediate post-exercise period. Most (n = 120, 94%) patients were in NYHA I-II functional class; 92 patients (72%) were on beta-blockers and 16 (13%) were on diuretic therapy. Eighteen patients (14%) had haemodynamically important LV outflow tract gradient (> 50 mm Hg) at baseline (**Table 1**). Twenty-three patients (18%) had moderate or severe MR at rest.

### LUS and exercise test findings

**Fig. 1:**
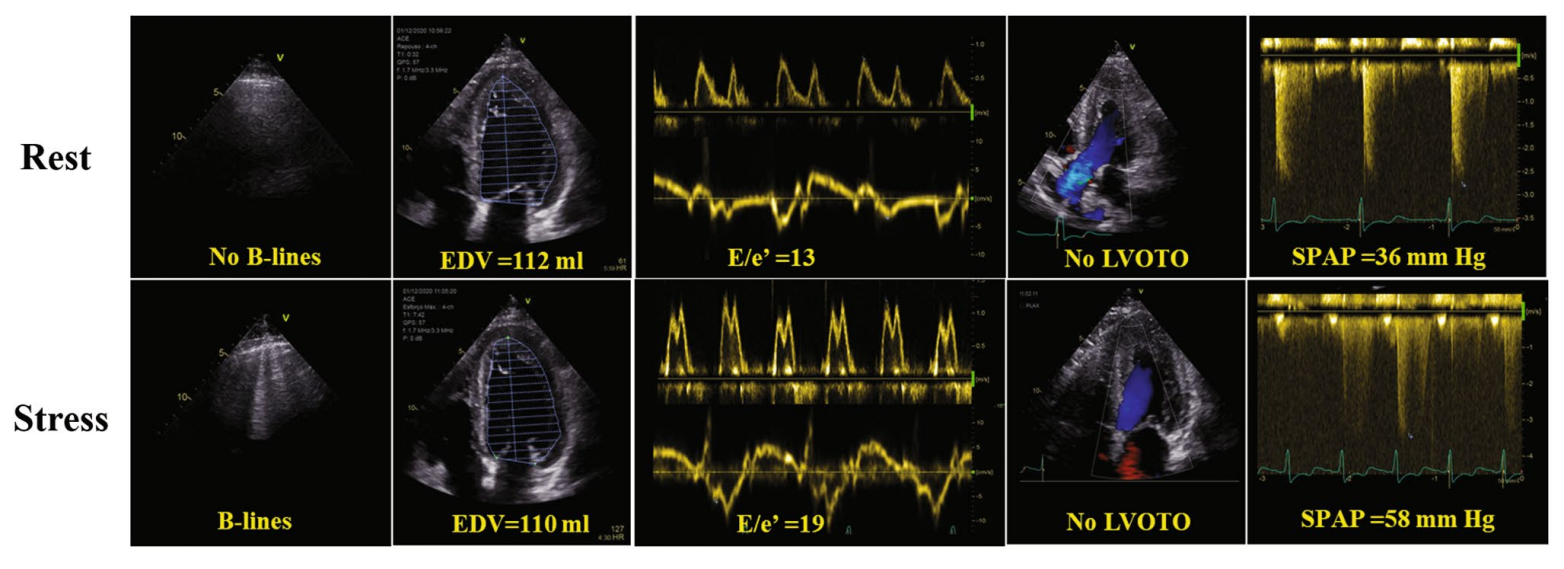
Example of exercise B-lines in a non-obstructive HCM patient with exertional dyspnea and negative coronary angiography. During ESE B-lines were associated with reduced diastolic reserve mirrored by falling EDV, increasing E/e’ and worsening pulmonary pressures. Abbreviations: E: early mitral inflow velocity; e’: early diastolic mitral annular velocity; EDV: end-diastolic volume; LVOTO: left ventricular outflow tract obstruction; SPAP: systolic pulmonary arterial pressure

No complications occurred during ESE. LUS was feasible in all subjects, with additional scanning and analysis time less than 1 min each for rest and peak stress. B-lines were detected in 13 patients at rest and in 38 during stress (12% vs. 31%, p < 0.0001). B-lines were present both at rest and at peak stress in 13 patients (12%). We divided the cohort into two groups according to the peak stress lung profiles: HCM patients with stress B-lines (congestive phenotype, with wet lungs: Group 1) and without stress B-lines (non-congestive phenotype, with dry lungs: Group 2). An example of LUS and ESE findings in a patient with B-lines is shown in Fig. 1. Exercise-time tended to be lower in patients with stress-induced B-lines (Group 1 = 8.7 ± 3.0 vs. Group 2 = 10.8 ± 3.8 min, p = 0.056). The reason for stopping the test was more frequently fatigue/exhaustion in patients with stress-induced B-lines (Group 1 = 54% vs. Group 2 = 32%, vs., p = 0.129). The second more frequent reason for prematurely stopping the test was dyspnea (Group 1 = 46% vs. Group 2 = 67%, p = 0.159).

### B-lines and clinical, echocardiographic and ESE findings

**Fig. 2:**
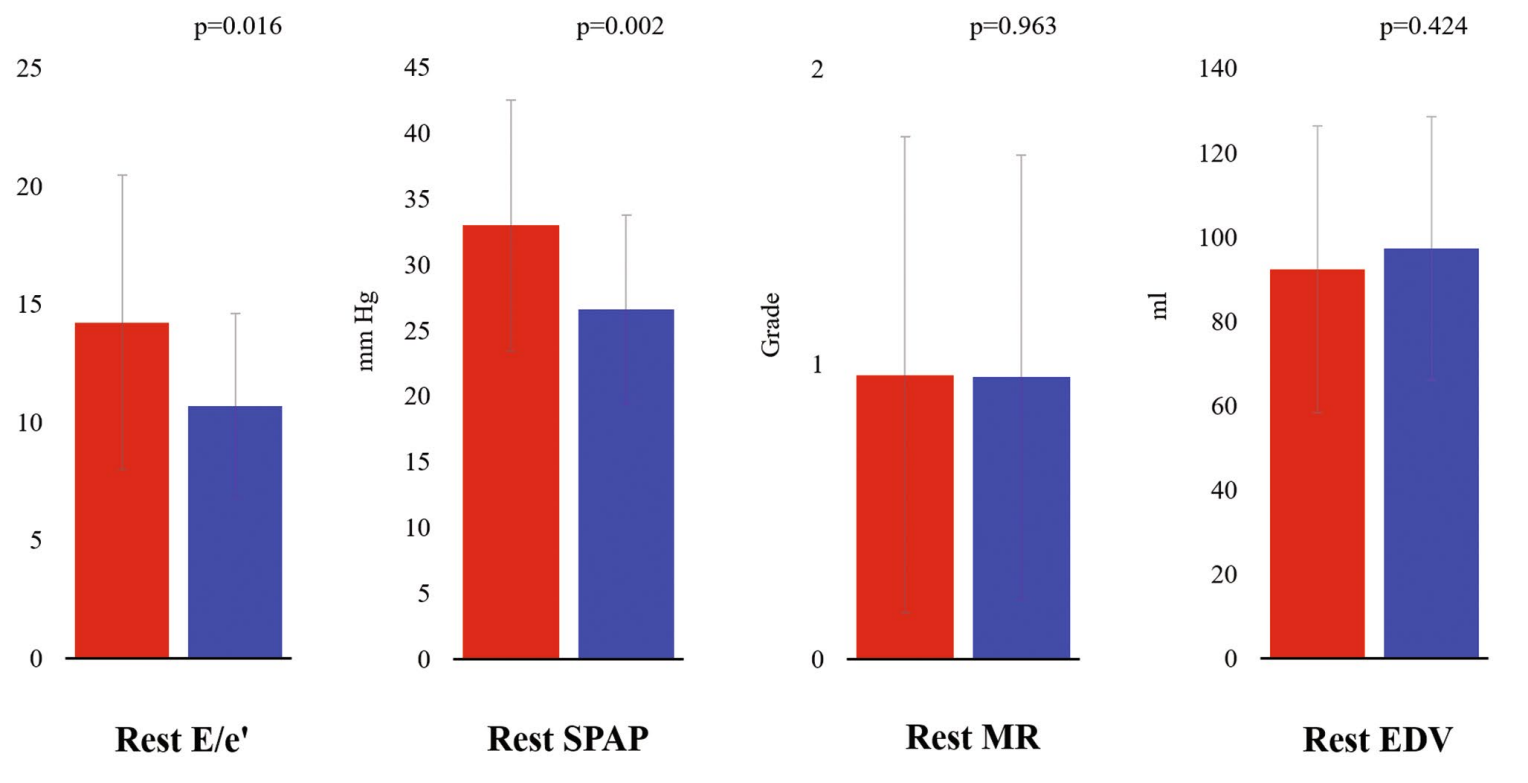
Stress B-lines and resting echocardiographic findings. From left to right: rest E/e’; rest SPAP; rest MR; rest EDV. Abbreviations as in Fig. 1. Red bar: Group 1 (with stress B-lines); Blue bar: Group 2 (without stress B-lines)

**Fig. 3:**
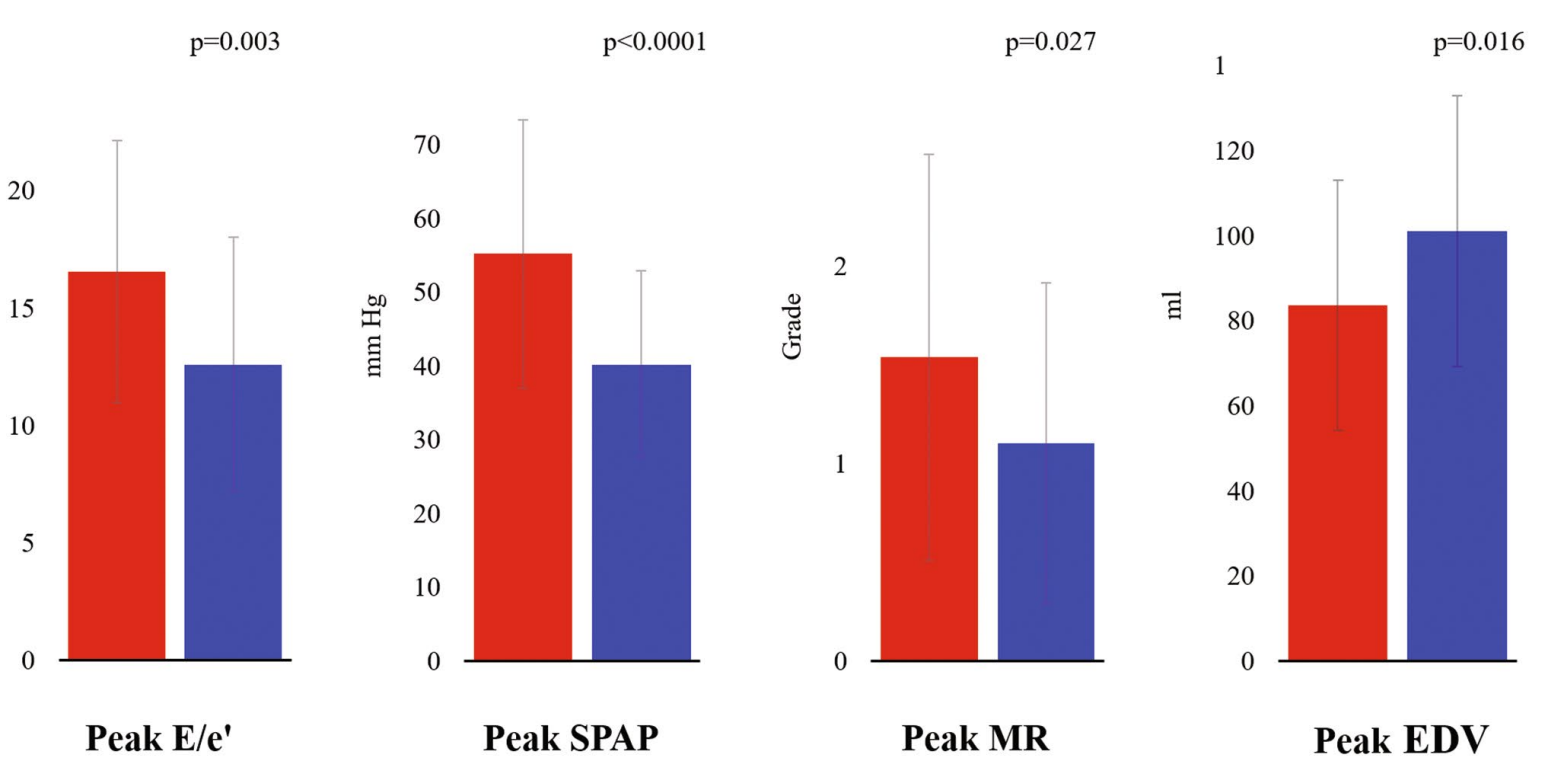
Stress B-lines and stress echocardiographic findings. From left to right: peak E/e’; peak SPAP; peak MR; peak EDV. Abbreviations as in Fig. 2. Red bar: Group 1 (with stress B-lines); Blue bar: Group 2 (without stress B-lines)

HCM patients in Group 1 were older at first diagnosis and had higher SCD risk scores at the time of the evaluation compared to patients in Group 2. At rest, patients with stress-B-lines showed a trend to higher prevalence of history of syncope (Group 1 = 11% vs. Group 2 = 4%, p = 0.236) but similar NYHA class than patients without stress B-lines (Table 1). In our population, 79% of “wet” patients with stress B-lines were off diuretic therapy, and 12% of “dry” patients without B-lines were on diuretic therapy. At rest, patients who developed stress B-lines had higher rest E/e’ and SPAP, with similar MR grade and EDV (Fig. 2). At peak stress, patients in Group 1 showed more elevated stress E/e’, SPAP, greater MR and smaller EDV compared to Group 2 patients (Table 2; Fig. 3). Another important finding was that patients in Group 1 showed twice more often a reduced preload response and an increase in MR in response to exercise (Table 2). Patients in Group 1 also showed lower baseline diastolic blood pressure, higher resting pulse pressure, more abnormal blood pressure response during exercise (47 vs 16%, p < 0.001) and lower stress SV index and CI (Table 3). The individual number of stress B-lines showed moderate positive correlation with peak exercise E/e’ (rs = 0.394 p < 0.001) and SPAP (rs = 0.326 p = 0.001) and inverse relationship with peak exercise SV index (rs=-0.359 p < 0.001) and CI (rs=-0.344 p < 0.001). Multivariate logistic regression analysis revealed that among baseline parameters the number of B-lines and SPAP were independent predictors of B-lines with exercise (Table 4).

**Table 1:** Baseline characteristics of the 128 HCM patients according to stress B-lines presence

	**All patients (n = 128)**			**HCM** **patients with stress B-lines (n = 38)**			**HCM** **patients without stress B-lines (n = 90)**			**p value**		
Age (years)	50.3	±	15.4	53.0	±	17.3	49.2	±	14.5	0.200		
**Age at first diagnosis (years)**	42.8	±	15.6	**48.4**	**±**	**17.8**	**40.9**	**±**	**14.5**	**0.031**		
Male gender	85 (66%)			24 (63%)			61 (68%)			0.613		
Body surface area (m^2^)	1.9	±	0.2	1.9	±	0.2	1.9	±	0.2	0.401		
**SCD risk (%)**	2.9	±	2.1	**4.1**	**±**	**3.2**	**2.6**	**±**	**1.5**	**0.039**		
Syncope	8 (6%)			4 (11%)			4 (4%)			0.236		
Coronary artery disease	4 (3%)			1 (3%)			3 (3%)			0.835		
NYHA I-II	120 (94%)			35 (92%)			85 (94%)			0.694		
Beta-blockers	92 (72%)			29 (76%)			63 (70%)			0.468		
Diuretics	16 (13%)			7 (18%)			9 (10%)			0.188		
LV max wall thickness (mm)	20.2	±	5.4	21.6	±	5.8	19.7	±	4.8	0.095		
LVOT gradient ≥30 mm Hg	26 (21%)			11 (29%)			15 (17%)			0.122		
LVOT gradient ≥50 mm Hg	18 (14%)			7 (18%)			11 (12%)			0.423		

Data are expressed as mean value ± SD, median value with the corresponding first and third quartile or number (%) of patients.

Abbreviations: HCM: hypertrophic cardiomyopathy; LV: left ventricular; LVOT: left ventricular outflow tract; NYHA: New York Heart Association; SCD: sudden cardiac death

**Table 2:** Rest and peak stress ultrasound findings according to stress B-lines presence in HCM (n = 128)

		**All patients** **(n = 128)**			**HCM** **patients** **with stress** **B-lines** **(n = 38)**			**HCM** **patients** **without** **stress B-lines** **(n = 90)**			**p value**		
**Lung ultrasound**													
**B lines score**	rest	0 (0;0)			0 (0;2)			0 (0;0)			**< 0.0001**		
	stress	0 (0;2)			4 (2;7)			0 (0;0)			**< 0.0001**		
**Echocardiographic parameters**													
LA diameter (mm)	rest	40.7	±	6.0	41.9	±	6.6	39.9	±	5.5	0.231		
LA volume (ml)	rest	69.0	±	28.1	79.4	±	38.5	65.9	±	23.6	0.107		
	stress	69.6	±	26.1	78.7	±	27.6	66.9	±	25.3	0.058		
	∆	-0.8	±	17.0	-3.8	±	19.7	0.0	±	16.1	0.342		
LAVi (ml/m2)	rest	36.9	±	14.8	41.2	±	18.9	35.6	±	13.2	0.176		
	stress	37.1	±	13.7	40.8	±	13.6	36.1	±	13.7	0.148		
	∆	-0.6	±	8.7	-1.9	±	9.6	-0.2	±	8.5	0.419		
**LV EDV (ml)**	rest	95.8	±	32.0	92.2	±	34.0	97.3	±	31.2	0.424		
	**stress**	97.2	±	32.0	**83.7**	**±**	**29.4**	**101.1**	**±**	**31.9**	**0.016**		
	∆	1.3	±	18.6	-4.8	±	18.8	3.1	±	18.3	0.064		
**Reduced preload response**		56 (44%)			**26 (68%)**			**30 (33%)**			**0.001**		
**LV EDVi (ml/m2)**	rest	51.0	±	17.2	48.4	±	17.7	52.0	±	17.0	0.293		
	**stress**	51.8	±	17.5	**43.4**	**±**	**14.1**	**54.2**	**±**	**17.7**	**0.006**		
	**∆**	0.8	±	10.5	-2.6	±	10.8	1.8	±	10.2	0.061		
LV ESV (ml)	rest	33.3	±	14.5	31.4	±	14.1	34.0	±	14.7	0.358		
	stress	30.6	±	16.7	27.8	±	13.3	31.4	±	17.6	0.340		
	∆	-2.7	±	13.4	-2.0	±	14.2	-3.0	±	13.2	0.751		
LV EF (%)	rest	65.4	±	7.8	65.9	±	8.0	65.2	±	7.8	0.632		
	stress	69.3	±	10.9	69.0	±	7.9	69.4	±	11.6	0.870		
LVOT gradient (mm Hg)	rest	21.5	±	27.7	25.7	±	33.8	19.7	±	24.7	0.321		
	stress	43.1	±	47.3	56.5	±	61.4	37.4	±	38.8	0.083		
	∆	21.6	±	30.7	30.7	±	42.7	17.7	±	23.1	0.084		
LV Force (mm Hg/ml)	rest	5.2	±	2.6	6.0	±	3.2	4.9	±	2.2	0.053		
	stress	8.6	±	5.1	10.8	±	6.9	8.0	±	4.4	0.066		
LVCR		1.7	±	0.7	1.8	±	0.8	1.7	±	0.6	0.591		
**MR (grade)**	rest	1.0	±	0.8	1.0	±	0.8	1.0	±	0.8	0.963		
	stress	1.2	±	0.9	1.5	±	1.0	1.1	±	0.8	0.057		
	**Δ**	0.2	±	0.6	**0.6**	**±**	**0.8**	**0.1**	**±**	**0.5**	**0.008**		
	**any change**	33 (26%)			**16 (42%)**			**17 (19%)**			**0.013**		
**SPAP (mm Hg)**	**rest**	27.8	±	8.0	**33.0**	**±**	**9.6**	**26.6**	**±**	**7.2**	**0.002**		
	**stress**	43.2	±	15.1	**55.2**	**±**	**18.2**	**40.2**	**±**	**12.6**	**< 0.0001**		
	**Δ**	15.4	±	12.9	**23.4**	**±**	**17.2**	**13.5**	**±**	**11.0**	**0.035**		
TAPSE (mm)	rest	23.9	±	4.5	24.2	±	5.4	23.8	±	4.3	0.714		
	stress	29.8	±	5.5	30.0	±	5.2	29.7	±	5.6	0.865		
	∆	5.8	±	4.9	5.6	±	5.4	5.9	±	4.8	0.854		
**E/e’**	**rest**	11.5	±	4.7	**14.2**	**±**	**6.2**	**10.7**	**±**	**3.9**	**0.016**		
	**stress**	13.5	±	5.7	**16.5**	**±**	**5.6**	**12.6**	**±**	**5.4**	**0.003**		
	∆	1.8	±	4.8	2.1	±	5.1	1.7	±	4.7	0.795		
WMSI	rest	1.0	±	0.1	1.0	±	0.0	1.0	±	0.1	0.389		
	stress	1.0	±	0.0	1.0	±	0.1	1.0	±	0.0	0.450		
	∆	0.0	±	0.1	0.0	±	0.1	0.0	±	0.1	0.199		
LV GLS (n = 67)	rest	-16.9	±	4.7	-17.1	±	4.9	-16.8	±	4.7	0.887		
Data are expressed as mean value ± SD, median value with the corresponding first and third quartile or number (%) of patients. Abbreviations: E: early mitral inflow velocity; e’: early diastolic mitral annular velocity; EDV: end-diastolic volume; EDVi: end-diastolic volume index; EF: ejection fraction; ESV: end-systolic volume; GLS: global longitudinal strain; HCM: hypertrophic cardiomyopathy; LA: left atrial; LAVi: left atrial volume index; LV: left ventricular; LVCR: left ventricular contractile reserve; LVOT: left ventricular outflow tract; MR: mitral regurgitation; SPAP: systolic pulmonary arterial pressure; TAPSE: tricuspid annular plane systolic excursion; WMSI: Wall motion score index													

**Table 3:** Rest and stress hemodynamic findings according to stress B-lines presence in HCM (n = 128)

		**All patients** **(n = 128)**			**HCM** **patients** **with stress** **B-lines** **(n = 38)**			**HCM patients without stress** **B-lines** **(n = 90)**			**p value**	
**Hemodynamic and exercise parameters**												
SBP (mmHg)	rest	125.1	±	15.4	128.4	±	15.6	123.7	±	15.1	0.110	
	stress	160.3	±	25.9	155.3	±	28.9	162.4	±	24.3	0.159	
	**∆**	35.2	±	27.4	26.9	±	30.6	38.7	±	25.4	0.025	
**DBP (mmHg)**	**rest**	78.0	±	9.5	**73.6**	**±**	**6.8**	**79.0**	**±**	**9.7**	**0.025**	
	stress	85.8	±	14.4	82.7	±	13.1	86.5	±	14.7	0.314	
	**∆**	7.8	±	13.7	9.1	±	14.9	7.5	±	13.4	0.640	
**Pulse pressure**	**rest**	46.5	±	13.9	**52.7**	**±**	**15.5**	**45.0**	**±**	**13.2**	**0.029**	
**(mmHg)**	stress	74.7	±	27.6	70.8	±	26.3	75.5	±	28.0	0.506	
	**∆**	28.2	±	28.3	18.1	±	26.1	30.5	±	28.4	0.085	
h (beats/min)	rest	67.6	±	14.6	66.2	±	10.0	68.2	±	16.2	0.485	
	stress	126.1	±	25.4	122.1	±	25.5	127.8	±	25.3	0.252	
	**∆**	58.5	±	24.7	55.9	±	25.7	59.6	±	24.4	0.445	
HHR		1.9	±	0.4	1.9	±	0.5	1.9	±	0.4	0.576	
**SV index**	rest	33.3	±	11.4	32.0	±	12.3	33.8	±	11.1	0.444	
**(ml/m** ^**− 2**^ **)**	**stress**	35.5	±	13.0	**29.1**	**±**	**10.6**	**37.3**	**±**	**13.1**	**0.005**	
	**Δ**	2.2	±	9.7	**-1.7**	**±**	**7.4**	**3.3**	**±**	**10.0**	**0.023**	
**Cardiac index (ml/min x m** ^**− 2**^ **)**	rest	2212	±	835	2070	±	732	2271	±	871	0.220	
	**stress**	4485	±	1797	**3474**	**±**	**1381**	**4778**	**±**	**1803**	**0.001**	
	**Δ**	2275	±	1465	**1530**	**±**	**1179**	**2492**	**±**	**1475**	**0.003**	
**ABPR**		32 (25%)			**18 (47%)**			**14 (16%)**			**< 0.001**	

Data are expressed as mean value ± SD or number (%) of patients

Abbreviations: ABPR: abnormal blood pressure response; DBP: diastolic blood pressure; HCM: hypertrophic cardiomyopathy; HR: heart rate; HRR: heart rate reserve; SBP: systolic blood pressure SV: stroke volume

**Table 4:** Baseline predictors of pulmonary congestion during exercise stress echocardiography in HCM (n = 128)

	**Univariate analysis**						**Multivariate analysis**					
	OR	95% CI			*p value*		OR	95% CI			*p value*	
Age at first diagnosis (years)	1.031	1.002	-	1.062	0.035							
SCD risk (%)	1.407	1.093	-	1.812	0.008							
**B lines number rest**	4.304	1.691	-	10.956	0.002		**4.282**	**1.120**	**-**	**16.364**	**0.033**	
DBP (mmHg)	0.932	0.876	-	0.993	0.029							
Pulse pressure (mmHg)	1.040	1.003	-	1.078	0.034							
LA volume (ml)	1.016	1.001	-	1.031	0.043							
E/e’ rest	1.154	1.049	-	1.271	0.003							
LV Force rest (mm Hg/ml)	1.183	1.019	-	1.373	0.027							
**SPAP rest (mm Hg)**	1.093	1.027	-	1.163	0.005		**1.070**	**1.000**	**-**	**1.144**	**0.048**	

OR: odds ratio; C.I.: confidence interval

Abbreviations as in Tables 1, 2 and 3

## Discussion

In the present study, LUS during ESE was feasible and simple in HCM, with 100% success rate for B-lines and only a minimal increase in imaging time. B-lines were found in about 10% of HCM patients at rest and in about 30% during ESE. HCM patients presenting B-lines at stress were diagnosed with HCM later in life and had higher SCD risk scores. They showed higher pulse pressure at rest, with similar heart rate and cardiac output compared to patients without stress B-lines, suggestive of a stiff aorta which may contribute to abnormal ventricular arterial interactions during stress eventually favouring myocardial fibrosis and dysfunction (15, 16). Stress B-lines were associated with worse diastolic function, greater SPAP and larger increment in MR during stress. Patients with pulmonary congestion at peak exercise had lower CI reserve at comparable heart rates, compared to those without B-lines, and more often had abnormal blood pressure response to exercise. Therefore, stress B-lines are relatively frequent findings in HCM patients, represent multiple mechanisms, are associated with signs of greater clinical and functional severity, and reflect hemodynamic vulnerability during exercise, mirrored by a reduced CI reserve and prevalent abnormal blood pressure response. Notably, the development of stress B-lines could not be reliably foreseen by the baseline echocardiographic features of our patients: the best predictor was SPAP > 28 mm Hg, with a positive predictive value of only 48%, 95% CI 32–65%. Therefore, given its ease of implementation and utility, the systematic performance of lung scanning appears a valuable adjunct to ESE in HCM patients, even in the presence of baseline evidence of increased pulmonary pressures. Albeit stress echocardiography is included in current guidelines for the management of HCM, mostly it is considered only a tool to evaluate peak LVOT gradients. However, ESE is a powerful multi-purpose tool with far-reaching clinical implications also in non-obstructive patients and provides much broader information for clinical practice.

### Pathophysiology of pulmonary congestion in HCM

The pathophysiology of heart failure and congestion in HCM is not yet completely understood (17). Left ventricular hypertrophy, ischemia and fibrosis lead to a stiff, non-compliant left chamber that restrains diastolic filling and elevates intracavitary end-diastolic pressures (1, 18). When the left ventricle fails, blood accumulates in the left atrium and left atrial pressure and pulmonary capillary pressure similarly rises (19). When pulmonary capillary pressure elevates above a threshold, the imbalance in the Starling forces across the pulmonary capillary endothelial barrier results in an increased accumulation of extravascular lung water (20). Distinct factors beyond diastolic dysfunction that contribute to backward HF in HCM include LVOT obstruction, structural or functional mitral valve alterations, increased large artery stiffness or less commonly, abnormal systolic function (21). Notably, baseline LVOTG was not a predictor of exercise pulmonary congestion. There was a trend of showing a higher gradients in the group of patients with stress B-lines but it was not significant. Although initially counterintuitive, this finding is consistent with clinical practice: only a minority of HCM patients with obstruction, even when severe, benefit from diuretics and many may worsen their symptoms due to preload reduction. Other factors seem to play a greater role than gradients, including the degree of MR at rest or during exercise and diastolic dysfunction.

### Comparison with previous studies

Numerous investigations have shown the excellent feasibility, diagnostic and prognostic usefulness of B-lines assessment during stress echocardiography in different cardiovascular diseases (7, 22, 23). However, our report is the first in the literature focusing on HCM. We adopted the simplified 4-site scan technique which proved to be the best trade-off between accuracy and simplicity both at rest and especially after stress when imaging time is short and there are many parameters to scan (7). Prior studies have demonstrated that the number of stress B-lines is tightly related to E/e’ and MR development during ESE in patients with HF, consistent with our findings in HCM (7, 23). In addition, we observed that in HCM stress B-lines were associated with lower EDV and CI reserve during stress. The findings of our study are in line with those of Lele et al., who evaluated 79 HCM outpatients in a hemodynamic study with radionuclide ventriculography and expiratory gas analysis during symptom-limited exercise stress. They found that the ability to increase left ventricular EDV is a principal factor in stroke volume and cardiac output augmentation during dynamic exercise in HCM (24). The advantage of ESE is that it provides a one-stop shop view of all these interconnected variables, including pulmonary congestion, preload reserve, dynamic intraventricular gradients and MR, both at rest and during stress.

### Clinical implications

Aggressive diuretic therapy can worsen symptoms related to LVOT obstruction by causing exaggerated decrease in preload and should be avoided in HCM. Conversely, with the clinical evidence of congestion, cautious use of low-dose diuretics can provide symptom relief and can be reasonable to apply also in patients with LVOT obstruction (3, 4). Clinical signs of pulmonary congestion such as pulmonary crackles on chest auscultation have substantial intra- and interobserver variability and are only loosely related to lung water accumulation (25). B-lines are also obtainable with pocket size instruments after a limited training and may guide an effective decongestion therapy with symptomatic and prognostic benefit, as it has been shown by randomized trials based on resting lung ultrasound in other clinical settings such as heart failure (26).

### Study limitations

We combined data from bicycle and treadmill ESE which have different hemodynamic effect and could have influence on cardiac volume changes and stress B-lines in some extent. Dynamic gradients are more obvious in orthostatic position, and treadmill increases EDV of the left ventricle more than semi-supine exercise in healthy subjects (11). Semi-supine exercise increases pulmonary artery wedge pressure more than upright exercise (27). Supine bicycle increases blood pressure more and heart rate less than treadmill, but thedouble product is similar (28). The observational study design did not interfere with the individual choice of the referring physician, which is a matter of personal experience, awareness of the individual patient indications and local practice. Data were obtained from different laboratories without core lab reading, but all readers underwent quality control prior to patient recruitment (8) and had established experience as referral centers for HCM. Transthoracic 2-dimensional echocardiography has recognized limitations in estimating absolute LV volumes in HCM but it remains the recommended first- line technique (29, 30). In the present study relative volumetric changes of EDV from rest to stress provided more information than absolute values. In assessing relative changes, most sources of inaccuracy average out and each patient acts as his or her own control during stress.

## Conclusions

LUS is feasible and easily accessible at rest and during ESE in HCM. Pulmonary congestion occurs in about 1 of 10 HCM patients at rest and in 1 of 3 during ESE. Diastolic impairment (mirrored by increased left ventricular filling pressures with reduced EDV reserve) and worsening of MR are main determinants of pulmonary congestion during exercise in HCM. The combination of ESE and LUS provides a dynamic assessment of the HCM pathophysiology and has the capability to recognize the pulmonary congestive phenotype, possibly useful for effective and personalized diuretic treatment, as it has been shown in heart failure patients without HCM. ESE with LUS may open a new diagnostic window for earlier and more precise detection of pulmonary congestion and diastolic dysfunction in HCM.

## Abbreviations

**CI**: cardiac index.
**EDV**: end-diastolic volume.
**EDV**: end-diastolic volume.
**EF**: ejection fraction.
**ESE**: exercise stress echocardiography LUS = lung ultrasound.
**ESV**: end-systolic volume.
**HCM**: hypertrophic cardiomyopathy.
**HR**: heart rate.
**LV**: left ventricle.
**LVOT**: left ventricular outflow tract.
**LVOTG**: left ventricular outflow tract gradient.
**MR**: mitral regurgitation.
**SBP**: systolic blood pressure.
**SCD**: Sudden cardiac death risk.
**SPAP**: systolic pulmonary arterial pressure.
**SV**: stroke volume.
**WMSI**: Wall motion score index.

## References

[1] Maron BJ, Maron MS, Olivotto I, Zipes DP, Peter L, Bonow RO, Mann DL, Tomaselli GF, Braunwald E (2019). Hypertrophic cardiomyopathy. Braunwald’s Heart Disease. A Textbook of Cardiovascular Medicine, Eleventh Edition.

[2] Rowin EJ, Maron BJ, Maron MS (2020 Sep) The Hypertrophic Cardiomyopathy Phenotype Viewed Through the Prism of Multimodality Imaging: Clinical and Etiologic Implications. JACC Cardiovasc Imaging. 13:2002–2016. 10.1016/j.jcmg.2019.09.020. Epub 2019 Dec 18. PMID: 31864978 9 10.1016/j.jcmg.2019.09.020 31864978

[3] Members WC, Ommen SR, Mital S, Burke MA, Day SM, Deswal A, Elliott P et al (2021 Jul) 2020 AHA/ACC guideline for the diagnosis and treatment of patients with hypertrophic cardiomyopathy: A report of the American College of Cardiology/American Heart Association Joint Committee on Clinical Practice Guidelines. J Thorac Cardiovasc Surg 162(1):e23–e106. doi: 10.1016/j.jtcvs.2021.04.001. Epub 2021 Apr 27. PMID: 33926766 10.1016/j.jtcvs.2021.04.001 33926766

[4] Elliott PM, Anastasakis A, Borger MA, Borggrefe M, Cecchi F, Charron P et al 2014 ESC Guidelines on diagnosis and management of hypertrophic cardiomyopathy: the Task Force for the Diagnosis and Management of Hypertrophic Cardiomyopathy of the European Society of Cardiology (ESC).Eur Heart J. 2014 Oct14;35(39):2733–79. doi: 10.1093/eurheartj/ehu284. Epub 2014 Aug 29. PMID: 25173338. 10.1093/eurheartj/ehu284 25173338

[5] Re F, Zachara E, Avella A, Baratta P, di Mauro M, Uguccioni M et al Dissecting functional impairment in hypertrophic cardiomyopathy by dynamic assessment of diastolic reserve and outflow obstruction: A combined cardiopulmonary-echocardiographic study.Int J Cardiol. 2017 Jan 15;227:743–750. doi: 10.1016/j.ijcard.2016.10.067. Epub 2016 Oct 28. PMID: 27839814. 10.1016/j.ijcard.2016.10.067 27839814

[6] Peteiro J, Barriales-Villa R, Larrañaga-Moreira JM, Bouzas-Mosquera A, Martinez-Veira C, Castro-Dios D et al (2021 May) Value of a comprehensive exercise echocardiography assessment for patients with hypertrophic cardiomyopathy. J Cardiol 77(5):525–531 Epub 2020 Dec 17. PMID: 33341337 10.1016/j.jjcc.2020.11.017 33341337

[7] Scali MC, Zagatina A, Ciampi Q, Cortigiani L, D’Andrea A, Daros CB et al (2020) Oct Stress Echo 2020 Study Group of the Italian Society of Echocardiography and Cardiovascular Imaging. Lung Ultrasound and Pulmonary Congestion During Stress Echocardiography. JACC Cardiovasc Imaging. ;13(10):2085–2095. doi: 10.1016/j.jcmg.2020.04.020. Epub 2020 Jul 15. PMID: 32682714. 10.1016/j.jcmg.2020.04.020 32682714

[8] Picano E, Ciampi Q, Citro R, D’Andrea A, Scali MC, Cortigiani L et al Stress echo 2020: the international stress echo study in ischemic and non-ischemic heart disease. Cardiovasc Ultrasound. 2017 Jan 18;15(1):3. doi: 10.1186/s12947-016-0092-1. PMID: 28100277; PMCID: PMC5242057 10.1186/s12947-016-0092-1 PMC5242057 28100277

[9] Pellikka PA, Arruda-Olson A, Chaudhry FA, Chen MH, Marshall JE, Porter TR et al (2020 Jan) Guidelines for Performance, Interpretation, and Application of Stress Echocardiography in Ischemic Heart Disease: From the American Society of Echocardiography. J Am Soc Echocardiogr 33(1):1–41e8 Epub 2019 Nov 15. PMID: 31740370 10.1016/j.echo.2019.07.001 31740370

[10] Lang RM, Badano LP, Mor-Avi V, Afilalo J, Armstrong A, Ernande L et al (2015) Jan;28(1):1–39.e14 Recommendations for cardiac chamber quantification by echocardiography in adults: an update from the American Society of Echocardiography and the European Association of Cardiovascular Imaging. J Am Soc Echocardiogr. doi: 10.1016/j.echo.2014.10.003. PMID: 25559473 10.1016/j.echo.2014.10.003 25559473

[11] Sicari R, Nihoyannopoulos P, Evangelista A, Kasprzak J, Lancellotti P, Poldermans D et al (2009) Feb;30(3):278 – 89 ; European Association of Echocardiography. Stress Echocardiography Expert Consensus Statement–Executive Summary: European Association of Echocardiography (EAE) (a registered branch of the ESC). Eur Heart J. doi: 10.1093/eurheartj/ehn492. Epub 2008 Nov 11. PMID: 19001473 10.1093/eurheartj/ehn492 19001473

[12] Bombardini T, Zagatina A, Ciampi Q, Arbucci R, Merlo PM, Haber DML et al (2021 Jun) Hemodynamic Heterogeneity of Reduced Cardiac Reserve Unmasked by Volumetric Exercise Echocardiography. J Clin Med 29(13):2906. doi: 10.3390/jcm10132906. PMID: 34209955; PMCID: PMC8267648 10.3390/jcm10132906 PMC8267648 34209955

[13] Lancellotti P, Pellikka PA, Budts W, Chaudhry FA, Donal E, Dulgheru R et al (2016) Nov;17(11):1191–1229 The clinical use of stress echocardiography in non-ischaemic heart disease: recommendations from the European Association of Cardiovascular Imaging and the American Society of Echocardiography. Eur Heart J Cardiovasc Imaging. doi: 10.1093/ehjci/jew190. Erratum in: Eur Heart J Cardiovasc Imaging. 2017 May 1;18(8):832. PMID: 27880640 10.1093/ehjci/jew190 27880640

[14] Ciampi Q, Zagatina A, Cortigiani L, Wierzbowska-Drabik K, Kasprzak JD, Haberka M et al Prognostic value of stress echocardiography assessed by the ABCDE protocol. Eur Heart J. 2021 Oct 1;42(37):3869–3878. doi: 10.1093/eurheartj/ehab493. PMID: 34449837; PMCID: PMC8486488 10.1093/eurheartj/ehab493 PMC8486488 34449837

[15] Roşca M, Mandeş L, Ciupercă D, Călin A, Beladan CC, Enache R et al Carotid arterial stiffness is increased and related to left ventricular function in patients with hypertrophic cardiomyopathy. Eur Heart J Cardiovasc Imaging. 2020 Aug 1;21(8):923–931. doi: 10.1093/ehjci/jez243. PMID: 31580440 10.1093/ehjci/jez243 31580440

[16] Chirinos JA, Segers P, Hughes T, Townsend R (2019) Large-Artery Stiffness in Health and Disease: JACC State-of-the-Art Review. J Am Coll Cardiol. Sep 3;74(9):1237–1263. doi: 10.1016/j.jacc.2019.07.012. PMID: 31466622; PMCID: PMC6719727 10.1016/j.jacc.2019.07.012 PMC6719727 31466622

[17] Maron BJ, Rowin EJ, Udelson JE, Maron MS (2018) May;6(5):353–363 Clinical Spectrum and Management of Heart Failure in Hypertrophic Cardiomyopathy. JACC Heart Fail. doi: 10.1016/j.jchf.2017.09.011. Epub 2018 Apr 11. PMID: 29655822 10.1016/j.jchf.2017.09.011 29655822

[18] Wigle ED, Sasson Z, Henderson MA, Ruddy TD, Fulop J, Rakowski H et al (1985) Jul-Aug;28(1):1–83 Hypertrophic cardiomyopathy. The importance of the site and the extent of hypertrophy. A review. Prog Cardiovasc Dis. doi: 10.1016/0033-0620(85)90024-6. PMID: 3160067 10.1016/0033-0620(85)90024-6 3160067

[19] Guyton A, Lindsey A Effect of elevated left atrial pressure and decreased plasma protein concentration on the development of pulmonary edema. Circ Res. 1959 Jul;7(4):649 – 57. doi: 10.1161/01.res.7.4.649. PMID: 13663218 10.1161/01.res.7.4.649 13663218

[20] West JB, Mathieu-Costello O (1992) Stress failure of pulmonary capillaries: role in lung and heart disease. Lancet. Sep 26;340(8822):762-7. doi: 10.1016/0140-6736(92)92301-u. PMID: 1356184 10.1016/0140-6736(92)92301-u 1356184

[21] Fifer MA, Baggish AL, Naidu SS (2015). Assessment of Heart Failure: Invasive and Non-invasive Methods. Hypertrophic Cardiomyopathy: Foreword by Bernard Gersh and Historical Context by Eugene Braunwald.

[22] Ciampi Q, Zagatina A, Cortigiani L, Wierzbowska-Drabik K, Kasprzak JD, Haberka M et al Prognostic value of stress echocardiography assessed by the ABCDE protocol. Eur Heart J. 2021 Oct 1;42(37):3869–3878. doi: 10.1093/eurheartj/ehab493. PMID: 34449837; PMCID: PMC8486488 10.1093/eurheartj/ehab493 PMC8486488 34449837

[23] Coiro S, Simonovic D, Deljanin-Ilic M, Duarte K, Carluccio E, Cattadori G et al (2020 Jun) Prognostic Value of Dynamic Changes in Pulmonary Congestion During Exercise Stress Echocardiography in Heart Failure With Preserved Ejection Fraction. Circ Heart Fail 13(6):e006769 Epub 2020 Jun 16. PMID: 32543975 10.1161/CIRCHEARTFAILURE.119.006769 32543975

[24] Lele SS, Thomson HL, Seo H, Belenkie I, McKenna WJ, Frenneaux MP Exercise capacity in hypertrophic cardiomyopathy. Role of stroke volume limitation, heart rate, and diastolic filling characteristics. Circulation. 1995 Nov 15;92(10):2886-94. doi: 10.1161/01.cir.92.10.2886. PMID: 7586256 10.1161/01.cir.92.10.2886 7586256

[25] Sherman RA (2016) Crackles and Comets: Lung Ultrasound to Detect Pulmonary Congestion in Patients on Dialysis is Coming of Age. Clin J Am Soc Nephrol. Nov 7;11(11):1924–1926. doi: 10.2215/CJN.09140816. Epub 2016 Sep 22. PMID: 27660304; PMCID: PMC5108202 10.2215/CJN.09140816 PMC5108202 27660304

[26] Mhanna M, Beran A, Nazir S, Sajdeya O, Srour O, Ayesh H et al (2021) Lung ultrasound-guided management to reduce hospitalization in chronic heart failure: a systematic review and meta-analysis. Heart Fail Rev. Apr 9. doi: 10.1007/s10741-021-10085-x. Epub ahead of print. PMID: 33835332 10.1007/s10741-021-10085-x 33835332

[27] Mizumi S, Goda A, Takeuchi K, Kikuchi H, Inami T, Soejima K et al (2018 Dec) Effects of body position during cardiopulmonary exercise testing with right heart catheterization. Physiol Rep 6(23):e13945. doi: 10.14814/phy2.13945PMID: 30548425; PMCID: PMC6289908 10.14814/phy2.13945 PMC6289908 30548425

[28] Badruddin SM, Ahmad A, Mickelson J, Abukhalil J, Winters WL, Nagueh SF et al (1999) May;33(6):1485-90 Supine bicycle versus post-treadmill exercise echocardiography in the detection of myocardial ischemia: a randomized single-blind crossover trial. J Am Coll Cardiol. doi: 10.1016/s0735-1097(99)00043-1. Erratum in: J Am Coll Cardiol 1999 Aug;34(2):613. PMID: 10334412 10.1016/s0735-1097(99)00043-1 10334412

[29] Nagueh SF, Bierig SM, Budoff MJ, Desai M, Dilsizian V, Eidem B, American Society of Echocardiography; American Society of Nuclear Cardiology; Society for Cardiovascular Magnetic Resonance; Society of Cardiovascular Computed Tomography (2011) May;24(5):473 – 98 ;. American Society of Echocardiography clinical recommendations for multimodality cardiovascular imaging of patients with hypertrophic cardiomyopathy: Endorsed by the American Society of Nuclear Cardiology, Society for Cardiovascular Magnetic Resonance, and Society of Cardiovascular Computed Tomography. J Am Soc Echocardiogr. doi: 10.1016/j.echo.2011.03.006. PMID: 21514501 10.1016/j.echo.2011.03.006 21514501

[30] Cardim N, Galderisi M, Edvardsen T, Plein S, Popescu BA, D’Andrea A et al Role of multimodality cardiac imaging in the management of patients with hypertrophic cardiomyopathy: an expert consensus of the European Association of Cardiovascular Imaging Endorsed by the Saudi Heart Association. Eur Heart J Cardiovasc Imaging. 2015 Mar;16(3):280. doi: 10.1093/ehjci/jeu291. Epub 2015 Feb 3. PMID: 25650407 10.1093/ehjci/jeu291 25650407

